# The measurement of health-related quality of life (QOL) in paediatric clinical trials: a systematic review

**DOI:** 10.1186/1477-7525-2-66

**Published:** 2004-11-22

**Authors:** Sally-Ann Clarke, Christine Eiser

**Affiliations:** 1 Department of Psychology, University of Sheffield, Western Bank, Sheffield, UK

## Abstract

**Background:**

The goal of much care in chronic childhood illness is to improve quality of life (QOL). However, surveys suggest QOL measures are not routinely included. In addition, there is little consensus about the quality of many QOL measures.

**Objectives:**

To determine the extent to which quality of life (QOL) measures are used in paediatric clinical trials and evaluate the quality of measures used.

**Design:**

Systematic literature review.

**Review Methods:**

Included paediatric trials published in English between 1994 and 2003 involving children and adolescents up to the age of 20 years, and use of a standardised QOL measure. Data Sources included MEDLINE, CINAHL, EMB Reviews, AMED, BNI, PSYCHINFO, the Cochrane library, Internet, and reference lists from review articles.

**Results:**

We identified 18 trials including assessment of QOL (4 Asthma, 4 Rhinitis, 2 Dermatitis, and single studies of Eczema, Cystic fibrosis, Otis media, Amblyopia, Diabetes, Obesity associated with a brain tumour, Idiopathic short stature, and Congenital agranulocytosis). In three trials, parents rated their own QOL but not their child's. Fourteen different QOL measures were used but only two fulfilled our minimal defined criteria for quality.

**Conclusions:**

This review confirms previous reports of limited use of QOL measures in paediatric clinical trials. Our review provides information about availability and quality of measures which will be of especial value to trial developers.

## Review

### Introduction

Chronic disease affects approximately 18% of children [1]. Although cure is not possible, survival rates have improved substantially for many conditions (e.g. cancer [2] and cystic fibrosis [3]). Many diseases require daily self-management and restrict children's physical and social activities. Consequently questions are increasingly raised about the quality of life (QOL) of children with chronic disease.

Efforts to measure child QOL have proved complex but a number of generic and disease-specific measures have been reported [4]. Generic measures are designed to assess and compare health status in patients with different diseases and may provide valuable information for comparing outcomes between sick and healthy populations. They are generally well validated and reliable but are often not recommended for work involving evaluation of randomised controlled trials (RCTs), as they lack sensitivity to detect small but clinically significant changes in QOL over time or due to treatment for specific diseases [5]. Disease specific measures are more suitable for evaluation of clinical trials designed to assess a particular treatment. These measures include items that are likely to be affected by the specific disease or treatment and are therefore more responsive to clinically significant changes.

The quality of measures must be evaluated according to performance characteristics. Guidelines suggest good measures of QOL are reliable and valid for the group of patients for whom they are used, include a form for self-report wherever possible, are brief and developmentally appropriate, and allow completion by proxy [4].

There is little evidence that QOL measures are routinely used in clinic practice [6] or clinical trials [7], despite the fact that the aim in many trials is to improve QOL. In both child [8] and adult work [9], few trials include measures of QOL, and amongst those, non-standardised measures continue to be used. QOL is also frequently insufficiently analyzed, reported or discussed in the study report or subsequent publications [5], despite the increasing emphasis in clinical practice and research to use patient centered outcomes and child perspectives [10].

We report a systematic review drawing on established methodologies [11] to determine first, the extent to which QOL measures are used in paediatric clinical trials and RCTs, and second, the quality of QOL measures currently used.

## Method

### Search Strategy

The following databases were searched: MEDLINE 1966 to Nov Week 2 2003, CINAHL 1982 to December Week 1 2003, EMB Reviews: Cochrane Central Register of Controlled Trials, Your Journals at OVID, EMB reviews: ACP Journal Club 1991 to July / August 2003, EMB reviews: Database of Abstracts and Reviews of Effects 3^rd ^quarter 1993, AMED (Allied & Contemporary Medicine) 1985 to December 2003, British Nursing Index (BNI) 1985 – October 2003, EMBASE, PSYCHINFO 1872–2003.

Text word and thesaurus searches were used to minimise the chance of missing relevant articles. The following keywords were searched:

• child, childhood, children, adolescent, infant, pediatric, paediatric,

• quality of life, QOL,

• clinical trial, randomised controlled trial

Searches were restricted to English language papers.

Search engines were used to search the Internet with keywords and Boolean logic. Additional references from articles identified through these searches were also pursued.

### Inclusion Criteria

These included:

1) Children and adolescents up to the age of 20 years,

2) RCT, formal cross-over trial, or studies evaluating one or more active drug treatment with or without placebo,

3) Standardised QOL measure (For these purposes we drew on a previous review [4] and defined minimal psychometric criteria to include some preliminary reliability and validity data),

4) Articles published in English between January 1994 and December 2003.

### Exclusion Criteria

1) Samples including both adults and children.

2) Comparison of surgical treatment, pain control, palliative medication, or psychological/homeopathic intervention.

3) Outcomes evaluated in terms of medical data only, non-standardised measures of QOL or standardised psychological measures including symptom checklists, measures of self-esteem, or coping.

### Procedure

Abstracts were reviewed for relevance and full articles obtained where appropriate. A summary sheet was developed and both authors independently reviewed papers to ensure reliability. Data extracted by reviewers was second coded and compared and any discrepancies were resolved through discussion.

## Results

Of the 917 records retrieved from the databases, initial inspection suggested that 27 abstracts met the inclusion criteria. On reading the full articles, nine failed to meet inclusion criteria. The resulting 18 articles were included in the review [12-29].

### Study characteristics (summarised in Table 1 [see Additional file 1])

• **Disease: **QOL was most frequently included in trials in atopic diseases (Asthma = 4, Rhinitis = 4, Dermatitis = 2 and Eczema = 1). Single studies were identified in Cystic fibrosis, Otis media, Amblyopia, Diabetes, Obesity associated with a brain tumour, Idiopathic short stature, and Congenital agranulocytosis.

• **Location: **Seven studies were conducted in the U.S.A, 4 in the U.K, 3 in the Netherlands, one in Taiwan, and one in Israel. Two studies were multi-national.

• **Child's age: **Three studies recruited children across a broad age range (1 to 18 years), 2 focused on pre-school children (1–5 years), 4 on pre-school and middle childhood (2–10 years), 2 on middle childhood (6–12 years), 6 on middle childhood and adolescence (5–18 years), and one on adolescents alone (12–17 years).

• **Sample size: **Sample size ranged from 19 [29] to 689 [15]. Power calculations were reported in six studies.

• **Design and trial aim: **We identified 11 RCTs, 2 cross-over studies, and 5 studies comparing two or more active treatments without placebo or control group. Of the 11 RCTs, 1 was multi-national, 7 multi centre, and 3 single centre studies. Of the 7 non RCTs, 1 was multi-national, 2 were multi centre, and 4 single centre. Nine articles involved comparisons of two or more treatment and the remainder involved comparison of treatments with placebo.

• **Blinding: **Seven RCTs reported blinding procedures.

• **Parent and caregiver QOL: **Fifteen studies measured the impact of the disease on the child's QOL. Three included assessment of the caregivers QOL.

• **Respondent for child QOL: **Of the 15 studies focusing on child QOL, 10 were based on child, and 3 on parent reports. In two studies both children and parents reported the child's QOL and in one of these clinicians also rated child QOL [28].

### Quality of QOL measures (Table 2 [see Additional file 2])

• **Generic or disease specific: **In total, 12 disease specific and two generic measures were used [30-38]. The four asthma trials involved three different measures of asthma specific QOL. The four perennial rhinitis trials used two different measures of rhinitis specific quality of life, and the two atopic dermatitis trials and one atopic eczema trial used two different dermatology specific measures of QOL. In two studies authors had developed their own disease specific measure [25,29].

• **Quality of measure: **We assessed quality of measures based on minimal accepted criteria [4] whereby measures should be brief, allow proxy and self report and include reliability and validity data and age appropriate versions. Although all measures included some preliminary psychometric data, only two measures fulfilled all of these criteria [36,38]. Three measures fulfilled four criteria but lacked age appropriate versions. The remaining measures fulfilled three or less criteria.

## Discussion

Despite extensive searches we identified only 18 published reports of paediatric trials including standardised QOL measures. This undoubtedly represents a very small percentage of paediatric trials and supports previous findings that QOL data is seldom reported in paediatric clinical trials [8]. Asthma and rhinitis were most frequently studied, perhaps because there is higher incidence for these conditions in children compared to other conditions such as cancer and cystic fibrosis [39]. Further explanations include the non-life threatening nature of these conditions as well as the availability of disease specific measures compared to rarer illnesses.

In considering why there are relatively few trials including QOL measures, it is important to take into account the aims and purpose of the trial [40,41]. The aim of most trials is to assess the impact of treatment on clinical variables, with QOL viewed to be of secondary importance if at all. It is not necessarily appropriate that QOL measures are included in all trials. Where QOL assessment is appropriate however, inclusion of a QOL measure must be hypothesis driven and an integral part of the clinical development programme rather than an added afterthought [5].

### Quality of measures

Where QOL was measured, disease specific measures were most often used (N = 12) as is normally recommended for use in clinical trials. Only two trials included measures that satisfactorily fulfilled accepted criteria [4]. Typically, information about measures included some reliability data although a third of studies failed to provide information about the validity of the scale. Most measures were brief and contained less than 30 items but many lacked age appropriate versions or parallel versions for child and proxy raters.

Selection of a measure of QOL is dependent on the psychometric properties of the instrument, as well as clinical and demographic variables characteristic of the sample. However, psychometric properties depend upon samples for which the scale has been validated. Hence it is important to ensure measures are used with clinical populations where psychometric data are available.

There are some grounds for assuming that QOL changes during childhood, and therefore satisfactory measures target specific age groups [42]. There are difficulties identifying single measures that are appropriate across a wide age range and only half of measures identified in this review included age appropriate versions.

It is also generally recommended that ratings of QOL should be made by children themselves whenever possible [43]. In cases of younger children proxy reports are necessary but there are questions about the relationship between child and parent report [4]. It is therefore positive that most (73.3%) studies obtained ratings from children with only four relying on parents alone to provide proxy ratings.

CONSORT [44] guidelines recommend methods of reporting RCTs, but do not adequately deal with the issues concerning QOL assessment and psychometric validity. It is essential that trial developers select appropriate measures and are aware of the problems associated with QOL assessment.

### Barriers to inclusion of QOL measures

Objections to inclusion of QOL measures in trials involve anticipated increased costs, extra time needed to gain patient and parent consent, and lack of sophistication of currently available measures [8]. A major restriction to inclusion of QOL assessment in clinical trials remains limitations in currently available measures, especially for less prevalent chronic conditions. However, it is only through including measures that we will learn more and be able to develop a second generation of measures that do show more sophisticated properties.

A second problem is that disease specific measures may simply not be available for rare conditions. Attempts to develop such measures are promising and in this review instruments for ambylopia [25] and agranulocytosis [29] had been developed. In order to facilitate collection of QOL data from children with chronic illness, reliable and valid measures are increasingly required [46].

Other methodological limitations in current work include the lack of power calculations. Where the aim of the trial includes QOL assessment, power calculations must be performed and are an essential element of clinical trial design. In cases where measurement of QOL is a secondary endpoint, sample size calculations are rare and difficult to establish. However attempts should be made to hypothesise expected changes in QOL scores in relation to the agreed sample size prior to the trial [5].

## Conclusion

This review supports previous findings of limited use of QOL measures in paediatric cancer trials [9] and extends this to include a number of conditions other than cancer. QOL assessment is most common in trials where the aim is to compare the impact of treatment on clinical variables and is largely limited to common non-life threatening conditions.

The measurement of QOL provides valuable information about the psychological and social impact of treatment on children especially where no differences in survival rates are anticipated. For this reason, the inclusion of QOL measurement in paediatric trials is becoming increasingly valued and mandatory [47,48]. There are still questions concerning selection of QOL measures and how best to report findings [49], but our review provides useful information for trial developers regarding the availability and quality of QOL measures.

## Author's contributions

Both authors were responsible for planning, conducting and reporting this work and approved the final manuscript.

## Supplementary Material

Additional File 1 **Table 1: Study characteristics** Click here for file

Additional File 2 **Table 2: Quality of life measures** Click here for file
