# Supplementary material for: The measurement of health-related quality of life (QOL) in paediatric clinical trials: a systematic review
Source: Health Qual Life Outcomes. 2004 Nov 22;2:66. doi: 10.1186/1477-7525-2-66 (PMC534785; doi:10.1186/1477-7525-2-66)
Supplement: Additional File 1 — Table 1: Study characteristics [file 1477-7525-2-66-S1.doc]

**Table 1: Study characteristics**

| Study | Objective of the trial | Study design | Age of child | Respondent | Outcomes |
| --- | --- | --- | --- | --- | --- |
| **Asthma** | | | | | |
| 12. Langton-Hewer, et al. (1995),  U.K. | To compare the efficacy of treatment (n = 11) with placebo (n = 12). | Randomised, double-blind, placebo- controlled. | 12-17 years. | Child QOL - child report. | No significant differences in QOL between treatment groups. |
| 13. Lemanske, et al. (2002),  Multi-centre, U.S.A. | To compare the efficacy of treatment (n = 225) with placebo (n = 109). | Randomised, double-blind, placebo-controlled. | 6-12 years. | Child QOL - child report. | Significant improvement in QOL in treatment group compared to placebo. |
| 14. Murphy, et al.(2003),  U.S.A. | To compare the efficacy of two treatments (n = 154, 133). | Randomised, open-label, parallel-group. | 2-6 years. | Caregiver QOL. | Significant improvement in QOL in one treatment group, and improvement in functional status of children in both groups. |
| 15. Knorr, et al. (2001),  Multi-centre (93)  Multi-national. | To compare the efficacy of treatment (n = 461) with placebo (n = 228). | Randomised, double-blind, placebo- controlled. | 2-5 years. | Caregiver QOL. | No significant differences in QOL between treatment groups. |
| **Perennial Rhinitis (allergic and non-allergic)** | | | | | |
| 16. Lai, et al. (2002),  Taiwan. | To compare the efficacy of three treatments with placebo (N = 80). | Randomised, double-blind, placebo-controlled. | 6-12 years. | Child QOL - child report | Significant improvement in QOL in two treatment groups. |
| 17. Milgrom, et al. (1999),  Multi-centre, U.S.A. | To compare the efficacy of two treatments (N = 71, 75). | Randomised, single-blind. No control group. | 6-18 years. | Child QOL - child report. | Significant improvement in QOL in both treatment groups. |
| 18. Meltzer, et al. (1997),  Multi-centre (6), U.S.A. | To compare the efficacy of treatment with placebo (N =204). | Randomised, double-blind, placebo -controlled, parallel group. | 6-18 years. | Child QOL - child report. | Significant improvement in QOL in treatment group compared to placebo. |
| 19. Fokkens, et al. (2002), Multi-centre, The Netherlands. | To compare the efficacy of treatment with placebo (N = 76). | Randomised, double-blind, placebo-controlled. | 6-16 years. | Child QOL - child report. | No significant differences  in QOL between treatment groups. |
| Atopic dermatitis | | | | | |
| 20. Whalley, et al. (2002),  Multi-centre (11),  U.S.A. | To compare the efficacy of two treatments (baseline N =241, 6 weeks N = 193, 6 months N = 161). | Two randomised, double blind, controlled, open label studies. | ≤ 8 years. | Parent QOL. | Significant improvement in QOL in both treatment groups. |
| 21. Harper, et al  (2000),  U.K. | To compare the efficacy of multiple short courses (n = 21) with continuous therapy (n = 19). | Randomised, open, parallel group. | 3-16 years. | Child QOL - child report. | Significant improvement in QOL in one treatment group. |

| Study | Objective of the trial | Study design | Age of child | Respondent | Outcomes |
| --- | --- | --- | --- | --- | --- |
| Atopic exzema | | | | | |
| 22. Thomas, et al (2002),  Multi-centre, U.K. | To compare the efficacy of two treatments (N = 207). | Randomised, double-blind, parallel group. | 1-15 years. | Child QOL - child report. | Clinically important improvements in QOL in both treatment groups. |
| **Cystic Fibrosis** | | | | | |
| 23. Suri, et al. (2002),  U.K. | To compare the efficacy and cost of two treatments (N = 48). | Randomised, open, crossover design. | 5-18 years. | Child QOL - parent report. | No significant differences in QOL between treatment groups. |
| **Persistent Otis Media with effusion (OME)** | | | | | |
| 24. Rovers, et al. (2001),  Multi-centre,  The Netherlands. | To compare the efficacy of treatment with control (N = 187). | Randomised, controlled. | 1-2 years. | Child QOL - parent report. | No significant differences in QOL between treatment group and control. |
| **Amblyopia** | | | | | |
| 25. Paediatric eye investigation group  (2003), Multi-centre (47), U.S.A. | To compare the efficacy of two treatments (N = 364, subset of 419). | Randomised, controlled. | < 7 years. | Child QOL - parent report. | One treatment correlated with improvement in QOL. |
| **Diabetes** | | | | | |
| 26. Weintrob, et al (2003), Israel. | To compare the efficacy of two treatments (N =  23) | Randomised, crossover design. | 9.4-13.9 years | Child QOL - child report. | No significant differences in QOL scores between treatment groups. |
| Hypothalamic obesity following cranial insult in child survivors of a brain tumour | | | | | |
| 27. Lustig, et al. (2003),  U.S.A. | 1) To compare the efficacy of treatment with placebo (N = 20). | Randomised, double blind, placebo-controlled. | 8-18 years. | Child QOL - child and parent report. | Treatment correlated with improvement in QOL. |
| **Idiopathic short stature (ISS)** | | | | | |
| 28. Theunissen et al (2002),  Multi-centre (3), The Netherlands. | 1) To compare the efficacy of treatment (n = 20) with control (n = 20). | Randomised, controlled. | 4-10 years. | Child QOL – child, parent, and clinician report. | Improved QOL reported by clinicians, no change reported by parents, and no change or deterioration reported by children. |
| Congenital agranulocytosis | | | | | |
| 29. Cleary, et al.  (1994),  Multi centre (11),  Multinational (4). | 1) To assess the efficacy of treatment (N = 19). | Phase 11 clinical trial. No control group or randomisation. | 4 1/2 months -18 years. | Child QOL - parent report. | Rg-CSF may result in significant improvements in general health perceptions, limitations of daily activities, and symptoms of the disease. |

n = number of children; N = Total sample
