# Supplementary material for: The measurement of health-related quality of life (QOL) in paediatric clinical trials: a systematic review
Source: Health Qual Life Outcomes. 2004 Nov 22;2:66. doi: 10.1186/1477-7525-2-66 (PMC534785; doi:10.1186/1477-7525-2-66)
Supplement: Additional File 2 — Table 2: Quality of life measures [file 1477-7525-2-66-S2.doc]

**Table 2: Quality of life measures**

| Measure (study) | Age range (years) | Child (self) report | Parent (proxy)  Report | Length | Reliability data | Validity data |
| --- | --- | --- | --- | --- | --- | --- |
| Disease specific measures of quality of life | | | | | | |
| **Asthma** | | | | | | |
| 30. CAQC (12) The Childhood Asthma Questionnaire  Christie, et al. (1993) | CAQA 4-7yrs  CAQB 8-11yrs  CAQC 12-16 yrs | ✓ | - | 22 items | ✓ | ✓ |
| 31. PAQLQ (13) Pediatric Asthma Quality of Life Questionnaire  Juniper, et al (1996) | 7-17yrs | ✓ | - | 23 items | ✓ | ✓ |
| 31. **PACQLQ** (14, 15)  Pediatric Asthma Caregivers Quality of Life Questionnaire, Juniper, et al (1996) | 7-17yrs | NA Caregiver QOL | NA | NA | ✓ | ✓ |
| **Perennial Rhinitis (allergic and non-allergic)** | | | | | | |
| **32**. **PRQLQ** **(16,19)**  **ARQLQ** (17,18,19)  Pediatric / Adolescent Rhinoconjunctivitis Quality of life Questionnaire, Juniper, et al (1993) | PRQLQ: 6-12yrs  ARQLQ: 12-17yrs | ✓ | - | 23 items  25 items | ✓ | ✓ |
| Atopic dermatitis & atopic eczema | | | | | | |
| 33. PIQol-AD (20) Parents index of Quality of life in atopic dermatitis Whalley, et al | < 8 yrs | NA  Parent QOL | NA | NA | ✓ | - |
| 34. CDLQI (21, 22) Children’s Dermatology Life Quality Index  Lewis-Jones & Finlay (1995) | 3-16yrs | ✓ | - | 10 items | ✓ | ✓ |
| **Amblyopia** | | | | | | |
| 25. **ATI** (25)  The Amblyopia Treatment Index  Paediatric eye investigation group (2003) | 3-6yrs | - | ✓ | 36 items | ✓ | ✓ |
| **Diabetes** | | | | | | |
| 35. DQOLY (26) Diabetes Quality of life Questionnaire for Youth, Ingersoll & Marrero (1991) | 11-18yrs | ✓ | - | 52 items | - | ✓ |
| **Hypothalamic obesity following cranial insult in child survivors of a brain tumour** | | | | | | |
| 36. **PCQL-32** version 1 (27)  Pediatric Cancer Quality of life  Varni, et al (1999) | 2-18  5-18 | ✓ | ✓ | 30 items | ✓ | ✓ |
| **Idiopathic short stature (ISS)** | | | | | | |
| 28. **ISSQOL** (28)  Idiopathic Short Stature Quality of Life | - | ✓ | ✓ | 8 items | ✓ | - |
| **Congenital Agranulocytosis** | | | | | | |
| 29. Authors developing measure specific to disease (29)  Cleary, et al (1994) | 4-18 yrs | - | ✓ | 26 items | ✓ | - |

| Measure (study) | Age range (years) | Child (self) report | Parent (proxy)  Report | Length | Reliability data | Validity data |
| --- | --- | --- | --- | --- | --- | --- |
| Generic Measures of quality of life | | | | | | |
| Cystic Fibrosis | | | | | | |
| 37. QWB (23) Quality of Well being Scale  Kaplan et al (1989) | 0-18 | ✓  adolescent only | ✓ | 23 items | ✓ | - |
| **Persistent Otis Media with Effusion (OME) and Idiopathic Short Stature (ISS)** | | | | | | |
| 38. **TNO-AZL** Quality of life questionnaires (24, 28, 28)  Verrips, et al (1999)  **TAIQOL** Infant  **TAQOL** Child  **DUCATQOL** Dutch Children’s | 1-4 yrs  6-12 yrs  5-16yrs | ✓ | ✓ | 35 items  -  - | ✓ | ✓ |
